# Supplementary material for: Enteric Pathogen Diversity in Infant Foods in Low-Income Neighborhoods of Kisumu, Kenya
Source: Int J Environ Res Public Health. 2019 Feb 12;16(3):506. doi: 10.3390/ijerph16030506 (PMC6388216; doi:10.3390/ijerph16030506)
Supplement: Supplementary file 1 [file ijerph-16-00506-s001.pdf]

Table S1. Taqman Array Card Primer and Probes.

Pathogen	Citation	Gene Target (25 characters max)	Forward Primer	Forward Primer #2	Reverse Primer	Probe Sequence	Probe Sequence #2
Adenovirus 40-41	1	Fiber Gene	AACTTTCTCTCTTAATAGACGCC		AGGGGGCTAGAAAACAAAA	CTGACACGGGCACTCT	
Adenovirus	2	Hexon	GCCACGGTGGGGTTTCTAAACTT		GCCCCAGTGGTCTTACATGCACATC	TGCACCAGACCCGGGCTCAG	
Astrovirus	2	Capsid	CAGTTGCTTGCTGCGTTCA		CTTGCTAGCCATCACACTTCT	CACAGAAGAGCAACTCCATCGC	
NorovirusGI	1	ORF 1-2	CGYTGGATGCGNTTYCATGA		CTTAGACGCCATCATCATTYAC	TGGACAGGAGATCGC	
NorovirusGII	2	ORF 1-2	CARGARBCNATGTTYAGRTGGATGAG		TCGACGCCATCTTCATTCACA	TGGGAGGGCGATCGCAATCT	
Rotavirus	2	NSP3	ACCATCTWCACRTRACCCTCTATGAG		GGTCACATAACGCCCCTATAGC	AGTTAAAAGCTAACACTGTCAAA	
Sapovirus	2	RdRp	GAYCAGGCTCTCGCYACCTAC	TTTGAACAAGCTGTGGCATGCTAC	CCCTCCATYTCAAACACTA	CYTGGTTCATAGGTGGTRCAG	CAGCTGGTACATTGGTGGCAC
EAEC	2	aaiC	ATTGTCCTCAGGCATTTCAC		ACGACACCCCTGATAAACAA	TAGTGCATACTCATCATTTAAG	
EAEC	2	aatA	CTGGCGAAAGACTGTATCAT		TTTTGCTTCATAAGCCGATAGA	TGGTTCTCATCTATTACAGACAGC	
EPEC	2	eae	CATTGATCAGGATTTTTCTGGTGATA		CTCATGCGGAAATAGCCGTTA	ATACTGGCGAGACTATTTCAA	
EPEC	2	bfpA	TGGTGCTTGCGCTTGCT		CGTTGCGCTCATTACTTCTG	CAGTCTGCGTCTGATTCCAA	
ETEC	2	LT	TTCCCACCGGATCACCAA		CAACCTTGTGGTGCATGATGA	CTTGGAGAGAAGAACCCT	
ETEC	2	STh	GCTAAACCAGYAGRGTCTTCAAAA		CCCGGTACARGCAGGATTACAACA	TGGTCCTGAAAGCATGAA	
ETEC	2	STp	TGAATCACTTGACTCTTCAAAA		GGCAGGATTACAACAAAGTT	TGAACAACACATTTTACTGCT	
STEC	2	Stx1	ACTTCTCGACTGCAAAGACGTATG		ACAAATTATCCCCTGWGCCACTATC	CTCTGCAATAGGTACTCCA	

STEC	2	Stx2	CCACATCGG TGTCTGTTAT TAACC	GGTCAAAAC GCGCCTGAT AG	TTGCTGTGGA TATACGAGG
EHEC _E_col i_0157	1	rfbE	TTTCACACTT ATTGGATGG TCTCAA	CGATGAGTTT ATCTGCAAG GTGAT	CTCTCTTTCCT CTGCGGTCCT
Aero monas	1	Aerolysin	TYCGYTACC AGTGGGACA AG	CCRGCAAAC TGGCTCTCG	CAGTTCCAGT CCCACCACTT
B_frag ilis	1	EGBF	GGGACAAGG ATTCTACCA GCTTTATA	ATTCGGCAAT CTCATTCATC ATT	CAATGGCGAA TCCATCAG
C_diff icile	2	tcdB	GGTATTACC TAATGCTCC AAATAG	TTTGTGCCAT CATTTTCTAA GC	CCTGGTGTCC ATCCTGTTTC
H_Pyl ori	1	ureC	GACACCAGA AAAAGCGGC TA	AGCGCATGT CTTCGGTTAA A	TCACTAAAGC GTTTTCTACC
Salmo nella_ enter	1	ttr	CTCACCAGG AGATTACAA CATGG	AGCTCAGAC CAAAAGTGA CCATC	CACCGACGGC GAGACCGACT TT
Shigell a- EIEC	2	ipaH	CCTTTTCCGC GTTCCTTGA	CGGAATCCG GAGGTATTGC	CGCCTTTCCG ATACCGTCTC TGCA
V_Ch olerae	1	hlyA	ATCGTCAGT TTGGAGCCA GT	TCGATGCGTT AAACACGAA G	ACCGATGCGA TTGCCCAA
V_par ahaem olytic	1	toxR	GTTTGGCGT GAGCAAGGT TT	AAGCGGGCT TAGGCGTTC	TCAAGCGATT TCTACTCTGC G
Crypt ospori dium	2	18s rRNA	GGGTTGTATT TATTAGATA AAGAACCA	AGGCCAATA CCCTACCGTC T	TGACATATCA TTCAAGTTTCT GAC
C_ho minus	1	LIB13	TCCTTGAAA TGAATATTTG TGACTCG	AAATGTGGT AGTTGCGGTT GAAA	CTTACTTCGTG GCGGCGT
C_par vum	1	LIB13	TCCTTGAAA TGAATATTTG TGACTCG	TTAATGTGGT AGTTGCGGTT GAAC	TATCTCTTCGT AGCGGCGTA
E_hist olytica	2	18s rRNA	ATTGTCGTG GCATCCTAA CTCA	GCGGACGGC TCATTATAAC A	TCATTGAATG AATTGGCCAT TT
Giardi a	2	18s rRNA	GACGGCTCA GGACAACGG TT	TTGCCAGCGG TGTCCG	CCCGCGGCGG TCCCTGCTAG
A_lu mbric oides	1	ITS1	GCCACATAG TAAATTGCA CACAAAT	GCCTTTCTAA CAAGCCCAA CAT	TTGGCGGACA ATTGCATGCG AT
N_am erican us	1	ITS2	CTGTTTGTCG AACGGTACT TGC	ATAACAGCG TGCACATGTT GC	CTGTACTACG CATTGTATAC

S_ster coralis	1	Dispersed repetitive sequence	TCCAGAAAA GTCTTCACTC TCCAG	TGCGTTAGAA TTTAGATATT ATTGTTGCT	TCAGCTCCAG TTGAACAACA GCCTCCAA
T_tric hiura	2	18s rRNA	TTGAAACGA CTTGCTCATC AACTT	CTGATTCTCC GTTAACCGTT GTC	CGATGGTACG CTACGTGCTT ACCATGG
MS2_	2	MS2g1	TGGCACTAC CCCTCTCCGT ATTCAC	GTACGGGCG ACCCCACGA TGAC	CACATCGATA GATCAAGGTG CCTACAAGC

References:
1. Liu J, Platts-Mills JA, Juma J, Kabir F, Nkeze J, Okoi C, Operario DJ, Uddin J, Ahmed S, Alonso PL, Antonio M, Becker SM, Blackwelder WC, Breiman RF, Faruque AS, Fields B, Gratz J, Haque R, Hossain A, Hossain MJ, Jarju S, Qamar F, Iqbal NT, Kwambana B, Mandomando I, McMurry TL, Ochieng C, Ochieng JB, Ochieng M, Onyango C, Panchalingam S, Kalam A, Aziz F, Qureshi S, Ramamurthy T, Roberts JH, Saha D, Sow SO, Stroup SE, Sur D, Tamboura B, Taniuchi M, Tennant SM, Toema D, Wu Y, Zaidi A, Nataro JP, Kotloff KL, Levine MM, Houpt ER. 2016. Use of quantitative molecular diagnostic methods to identify causes of diarrhoea in children: a reanalysis of the GEMS case-control study. Lancet 388:1291-301.
2. Liu J, Gratz J, Amour C, Kibiki G, Becker S, Janaki L, Verweij JJ, Taniuchi M, Sobuz SU, Haque R. 2013. A laboratory-developed TaqMan Array Card for simultaneous detection of 19 enteropathogens. Journal of clinical microbiology 51:472-480.

Inhibition and MS2 Extraction Efficiency of ZymoBIOMICS™ DNA/RNA Extraction Mini-kit.

Among the food samples that were tested for inhibition, only 1% were positive for inhibition (1/77). One-way ANOVA showed that Ct values of field spiked MS2 virus were not statistically different between 77 food samples plus the 10 water-only controls (p-value= 0.53) and between individual food types (p-value = 0.47) (Table S2). The lack of inhibition and high extraction efficiency in recovery of MS2 indicated that the DNA and RNA extracted from food was of good quality and was not biased by food physical properties.

Table S2. MS2 Ct Values Across Food Type.

Food Type	Number of Samples	Mean CT	Standard Deviation
Water only field control	10	13.0	0.6

Milk	11	13.2	1.3
Porridge	53	12.7	0.9
Tea	3	12.5	0.3
Water	10	13.1	1.6
